# Severe, Unremitting Piriformis Syndrome Following Yoga and Pilates: A Case Report

**DOI:** 10.7759/cureus.99504

**Published:** 2025-12-17

**Authors:** Abdullatif S Ibrahim, Sameera Hajijama, Nezar Albar, Fatima B Athar, Hamza A Shikh

**Affiliations:** 1 College of Medicine, Batterjee Medical College, Jeddah, SAU; 2 Medicine, Mohammed Bin Rashid University of Medicine and Health Sciences, Dubai, ARE; 3 Medicine, Dr. Samir Abbas Hospital, Jeddah, SAU; 4 College of Medicine, Alfaisal University, Riyadh, SAU; 5 College of Medicine, Ibn Sina National College for Medical Studies, Jeddah, SAU

**Keywords:** pilates-related injury, piriformis syndrome, sciatic nerve entrapment, sport-related pain exacerbation, yoga-induced musculoskeletal pain

## Abstract

Piriformis syndrome (PS) is caused by entrapment of the sciatic nerve due to spasm of the piriformis muscle, resulting in debilitating pain and discomfort. It is challenging to diagnose clinically, particularly when influenced by unusual triggers such as yoga or Pilates. Although anatomical factors and physical strain are commonly attributed to the etiology of the condition, studies have shown that psychological or environmental influences may contribute to the development and persistence of symptoms. This case report explores the atypical management of severe PS and the use of an integrated approach to sport-related pain exacerbations. We report a 60-year-old woman with a history of severe anxiety disorder who developed PS following yoga and Pilates sessions. The patient’s pain remained severe and unremitting despite receiving standard therapy. She began to show clinical improvement only after her duloxetine dose was increased back to 60 mg and amitriptyline was added, with full pain recovery occurring three weeks thereafter. By prioritizing this integrated approach, the patient noted a rapid reduction in pain intensity and an improvement in overall symptoms. She continued to improve on the medication regimen with no recurrence of sport-related pain exacerbations. The use of psychiatric medication for pain management in patients with PS or other sport-related injuries must be investigated further. This case underlines the importance of addressing holistic factors in managing musculoskeletal pain syndromes.

## Introduction

Piriformis syndrome (PS) is a condition characterized by the compression or irritation of the sciatic nerve due to the piriformis muscle, resulting in debilitating pain and discomfort [1]. Although anatomical factors and physical strain are commonly attributed to this syndrome, psychosomatic influences may possibly contribute to the development and persistence of symptoms [2,3]. Activities such as yoga and Pilates, which often involve eccentric contraction of the gluteal muscles or extreme ranges of hip motion (external rotation and abduction), can predispose individuals to piriformis muscle irritation and subsequent sciatic nerve compression. Anxiety may perpetuate symptoms through mechanisms such as central sensitization, increasing muscle tension, and amplifying pain perception.

This case is notable due to the combination of severe piriformis-related pain that was refractory to conventional and invasive interventions (including medications, physiotherapy, and local injections), concurrent psychological stressors, and a marked response to targeted antidepressant therapy, illustrating the complex biopsychosocial interplay in symptom management. This case presentation explores the detailed journey of a patient suffering from severe and unremitting PS in the context of an underlying severe anxiety disorder. We report a unique case of piriformis muscle syndrome, highlighting its clinical features, diagnostic approach, and management strategy.

This case was previously presented as an oral presentation at the 38th FIMS World Congress of Sports Medicine - Dubai, held on October 24, 2024, underscoring its relevance to sports medicine and musculoskeletal practice.

## Case presentation

A 60-year-old female patient presented with severe right gluteal pain following three consecutive sessions of yoga and Pilates, an exercise regimen she had recently adopted after being inactive for nearly a year. The pain extended to the back of her thigh and crossed the knee and was 8-9/10 in severity. Her medical history included a decade-long history of severe anxiety disorder, well-controlled on duloxetine 60 mg for 10 years. The dose had been gradually tapered at her request, as she had remained stable and asymptomatic for years, and she was taking 48 mg at the time of presentation. She is also diagnosed with pre-diabetes, has had four cesarean sections, and is an occasional smoker.

Clinical examination noted severe right gluteal pain that radiated down her right leg, following the path of the sciatic nerve crossing the knee, leading to the clinical diagnosis of the condition. Specifically, the Freiberg test and other relevant maneuvers were performed and elicited reproducible pain consistent with PS. Conventional physiotherapy was initiated but failed to provide any noticeable improvement over time. Her initial medication regimen included pregabalin, ibuprofen, paracetamol, celecoxib, B12, and B1 with alpha-lipoic acid. Physiotherapy and medication failed to control the pain for over two weeks.

Frustrated by her unrelenting pain, she sought further evaluation at a pain clinic. Lumbar spine X-ray and MRI were performed during this period, which were normal and showed no evidence of disc herniation or nerve root compression, ruling out lumbar radiculopathy as a cause of her symptoms. Botox, hydrocortisone, and lidocaine injections into and around the piriformis muscle gave pain relief for only one day. Following that comfort, she continued to use a second dose of steroid injections (dexamethasone), leading to 50% relief according to the patient. However, that relief only lasted for three days, and afterwards the pain re-emerged with an even greater intensity. Consequently, she sought care at another pain clinic, where she underwent multiple injections targeting the facet joints of the lower lumbar spine and pelvic girdle. These were administered as part of an evaluation for possible lumbar pathology, which was ultimately ruled out, and the injections provided only partial relief lasting about one week.

Despite multiple invasive methods of treatment, the patient’s pain persisted with little improvement. Throughout this challenging period, the patient's psychological distress and anxiety became increasingly evident. This psychological component was found to be significantly exacerbating her pain. Her management plan was then consequently revised and improved to incorporate an increased duloxetine dose (60 mg) and the introduction of amitriptyline (10 mg).

Remarkably, this change in her medication regimen coincided with a rapid reduction in pain intensity. Within seven days of returning to duloxetine 60 mg and starting amitriptyline 10 mg, she achieved complete pain recovery, regained full range of motion, and was able to tolerate physical therapy, with her pain score on the Visual Analog Scale (VAS) decreasing from 8/10 at presentation to 0/10. Her medications were reduced, including a gradual tapering of pregabalin and minimal use of as-needed oxycodone, in conjunction with celecoxib thrice daily. An equally noteworthy consequence was the patient's improved mood and enhanced pain control, ultimately eliminating her reliance on oxycodone.

Table 1 summarizes the interventions used in this case and the corresponding patient responses, including duration and effectiveness.

**Table 1:** Summary of Interventions and Patient Responses VAS: Visual Analog Scale

Intervention	Details/Dosage	Outcome/Duration
Physiotherapy	Conventional sessions	No improvement over two weeks
Initial medications	Pregabalin, ibuprofen, paracetamol, celecoxib, B1, and B12 with alpha-lipoic acid	Pain uncontrolled for over two weeks
Piriformis injections	Botox + hydrocortisone + lidocaine	Relief for one day
Second piriformis injection	Dexamethasone	50% relief, lasted three days
Facet joint injections	Lower lumbar spine and pelvic girdle; lumbar X-ray/MRI normal	Partial relief around one week; lumbar pathology ruled out
Optimized medications	Duloxetine 60 mg; amitriptyline 10 mg	Complete pain recovery within seven days; VAS 8 → 0; full range of motion; able to tolerate physical therapy; gradual taper of pregabalin; minimal as-needed oxycodone; celecoxib TID

## Discussion

PS is considered an entrapment neuropathy, in which the sciatic nerve is compressed within the piriformis muscle. The pathophysiology of this condition is not precisely defined, but it is believed to share similar etiologies with other causes of sciatic nerve impingement. Common contributing factors include trauma to the hip or buttocks, prolonged sitting, or muscle strain that leads to scarring and fibrosis around the nerve [4,5]. Patients may also develop buttock pain during downhill running or sprinting, as the piriformis muscle undergoes eccentric contraction during these movements, which in some runners may trigger the syndrome [6]. Another important consideration is anatomical variation, such as a bifid piriformis muscle or branching variations of the sciatic nerve as it passes through or around the muscle [7,8]. The etiologies of PS may overlap with other causes of sacroiliac joint pain or muscle syndromes; thus, ruling out alternative diagnoses is essential.

Diagnosis of PS is primarily clinical and supported by provocative maneuvers. The Freiberg test, performed by placing the hip in extension and internal rotation while resisting external rotation, is considered positive if pain or sciatic symptoms are reproduced [9]. In this patient, the test was positive, eliciting severe buttock pain. Other commonly used maneuvers include the flexion-adduction-internal rotation (FADIR) test, pace test, and Beatty test [10]. Imaging such as ultrasound, CT, or MRI is generally used to exclude other causes of hip or buttock pain, such as lumbar strain, disc herniation, or spinal stenosis [4]. Preliminary studies suggest that ultrasound assessment of piriformis muscle thickness and cross-sectional area may provide additional diagnostic value [11].

First-line treatment typically involves physical therapy focused on stretching, relieving piriformis tension, and strengthening the pelvic and hip region. Most patients respond well to conservative measures [6]. Analgesics such as nonsteroidal anti-inflammatory drugs (NSAIDs) or muscle relaxants may be used as needed. Ultrasound-guided glucocorticoid injections have been beneficial in selected cases [12], and botulinum toxin injections have been attempted, though their efficacy is often short-lived [13]. In rare cases of severe, refractory symptoms, surgical options such as piriformis tenotomy may be considered [14].

The association between PS and psychiatric conditions is poorly studied. Some evidence links PS with fibromyalgia [15,16], and there are reports of patients with mental health disorders presenting with nonspecific muscle pain [17,18]. However, no studies directly emphasize psychiatric comorbidities as triggers for the initial presentation of PS. This highlights the importance of case reports in raising awareness about such phenomena.

The role of antidepressants in managing PS also warrants further exploration. In our case, the patient’s symptoms improved significantly after optimizing her antidepressant therapy. The mechanism of improvement may involve both the pharmacologic effects of the serotonin-norepinephrine reuptake inhibitors (SNRIs) and tricyclic antidepressants (TCAs) on neuropathic pain, as well as the reduction of anxiety-induced muscle tension. However, this requires further study, particularly as the patient was experiencing significant social stressors at the time of symptom onset. While her pain improved rapidly, her anxiety persisted and required ongoing management with psychotherapy over the following six to eight months. A large meta-analysis of 33 trials including 5,318 participants evaluated the use of antidepressants for other causes of lower back pain, including sciatica and osteoarthritis. Moderate-certainty evidence demonstrated that SNRIs reduced back pain (mean difference -5.30, 95% confidence interval (CI) -7.31 to -3.30) at 3-13 weeks, with low-certainty evidence suggesting benefit in osteoarthritis pain (-9.72, 95% CI -12.75 to -6.69) at 3-13 weeks. Very low-certainty evidence indicated that SNRIs might reduce sciatica at two weeks or less (-18.60, 95% CI -31.87 to -5.33), but not consistently at 3-13 weeks (-17.50, 95% CI -42.90 to 7.89). Moderate-certainty evidence also showed that SNRIs reduced disability from back pain at 3-13 weeks (-3.55, 95% CI -5.22 to -1.88) and disability due to osteoarthritis at two weeks or less (-5.10, 95% CI -7.31 to -2.89), with low-certainty evidence at 3-13 weeks (-6.07, 95% CI -8.13 to -4.02). In contrast, TCAs and other classes of antidepressants did not significantly reduce pain or disability from back pain. This finding is particularly noteworthy, as our patient improved with the addition of amitriptyline (a TCA) and an increased dose of duloxetine (an SNRI).

While the patient demonstrated remarkable improvement following the optimization of duloxetine and initiation of amitriptyline, it is important to note that the evidence supporting the use of SNRIs and TCAs specifically for PS is limited. These medications are well-established for neuropathic pain and central sensitization syndromes, and their use in this case was guided by this mechanism, rather than direct evidence for peripheral nerve entrapment outside of conditions like diabetic neuropathy. PS involves both mechanical compression and potential neuropathic amplification; therefore, pharmacologic therapy likely targeted the latter component. Additionally, the patient had recently ceased invasive interventions, which themselves may have caused transient pain flare-ups. Consequently, the observed improvement cannot be attributed solely to the medications, and this potential confounder should be considered when interpreting treatment outcomes.

## Conclusions

This case report highlights the complex interplay between musculoskeletal pain, such as PS, and comorbid psychological conditions, including anxiety. The timely and carefully calibrated optimization of psychotropic medications, including duloxetine and amitriptyline, was likely a key turning point in controlling pain and improving the patient’s overall well-being within a multimodal treatment approach. This case underscores the value of a comprehensive management strategy that addresses both physical and psychosomatic factors. While it cannot definitively establish that psychological distress was the primary driver of pain, it illustrates the dual benefit of using SNRIs and TCAs in patients with neuropathic or entrapment pain who also have coexisting anxiety, reinforcing the importance of holistic and multidisciplinary care.
